# Hepatitis C Virus Infection and Hospital-Related Outcomes: A Systematic Review

**DOI:** 10.1155/2024/3325609

**Published:** 2024-03-07

**Authors:** Michelle Ng, Patrizia Maria Carrieri, Lindila Awendila, Maria Eugenia Socías, Rod Knight, Lianping Ti

**Affiliations:** ^1^British Columbia Centre on Substance Use, 400-1045 Howe Street, Vancouver, British Columbia, Canada V6Z 2A9; ^2^Faculté de Médecine, Aix Marseille Université, 27 bd Jean Moulin 13385, Marseille Cedex 5, France; ^3^British Columbia Centre for Excellence in HIV/AIDS, 608-1081 Burrard St, Vancouver, British Columbia, Canada V6Z 1Y6; ^4^Department of Medicine, University of British Columbia, 2775 Laurel Street, Vancouver, British Columbia, Canada V5Z 1M9; ^5^École de Santé Publique de l'Université de Montréal, 7101 Avenue du Parc, Montréal, Québec, Canada H3N 1X9

## Abstract

**Background:**

People living with hepatitis C infection (HCV) have a significant impact on the global healthcare system, with high rates of inpatient service use. Direct-acting antivirals (DAAs) have the potential to alleviate this burden; however, the evidence on the impact of HCV infection and hospital outcomes is undetermined. This systematic review aims to assess this research gap, including how DAAs may modify the relationship between HCV infection and hospital-related outcomes.

**Methods:**

We searched five databases up to August 2022 to identify relevant studies evaluating the impact of HCV infection on hospital-related outcomes. We created an electronic database of potentially eligible articles, removed duplicates, and then independently screened titles, abstracts, and full-text articles.

**Results:**

A total of 57 studies were included. Analysis of the included studies found an association between HCV infection and increased number of hospitalizations, length of stay, and readmissions. There was less consistent evidence of a relationship between HCV and in-hospital mortality. Only four studies examined the impact of DAAs, which showed that DAAs were associated with a reduction in hospitalizations and mortality. In the 14 studies available among people living with HIV, HCV coinfection similarly increased hospitalization, but there was less evidence for the other hospital-related outcomes.

**Conclusions:**

There is good to high-quality evidence that HCV negatively impacts hospital-related outcomes, primarily through increased hospitalizations, length of stay, and readmissions. Given the paucity of studies on the effect of DAAs on hospital outcomes, future research is needed to understand their impact on hospital-related outcomes.

## 1. Introduction

Chronic hepatitis C virus (HCV) infection constitutes a major public health challenge, with an estimated 71 million people affected worldwide [1, 2]. People living with HCV have a significant impact on the global healthcare system, with high utilization rates of inpatient services [3, 4]. Between 2005 and 2014, hospitalizations involving HCV increased by 59.8% in the United States (US) and were associated with increased length of stay, a rise in average costs, and higher in-hospital mortality when compared to stays without HCV [5]. While it appears that traditional liver complications such as variceal bleeding and ascites have remained stable in recent years, nonliver complications such as infection (e.g., pneumonia, cellulitis, and urinary tract infections) and renal failure have increased among those hospitalized, perhaps reflecting the high proportion of comorbidities among those with HCV [6]. With inpatient services being responsible for two-thirds of the total economic costs of HCV, it is expected that the burden of chronic HCV infection on hospital systems will continue to be significant [7, 8].

The increasing burden of acute inpatient care for HCV is largely among those born between 1945 and 1965 [4, 5, 9]. According to US surveillance data, HCV-related hospitalizations were the highest among this “baby boomer” generation and 3 to 4 times the rate of other age groups [5]. Furthermore, hospitalizations in this group increased by 67.3% between 2005 and 2014, while they decreased in younger people living with HCV [5]. A large proportion of people with HCV tested positive prior to rigorous blood donation screening and are now of older age [10]. Advancing age and increased duration of infection parallel the slow onset and progression of HCV-related complications such as advanced liver disease [10–12].

Direct-acting antivirals (DAAs) have made widespread treatment of hepatitis C infection a feasible strategy [2, 13–15]. With high cure rates and proven efficacy in various populations, DAAs are an appealing approach to control this global epidemic and its expanding financial burden on acute care systems [8, 16]. An observational study in Italy found that antiviral treatment of HCV decreased hospitalization costs from 85% to 10% of total healthcare expenditure [8]. However, as of yet, there has not been a comprehensive review of the impact of HCV infection on hospital-related outcomes and how DAAs could influence these outcomes. While there is literature from the US suggesting increased hospitalizations in those with HCV infection, there has not been a review done in a systematic manner on a global scale. Additionally, there has been no previous systematic review or meta-analysis examining the role of HCV on hospital-related outcomes beyond hospitalization. Therefore, the purpose of this review was to comprehensively assess the literature to better understand the impact of HCV on hospital-related outcomes, including hospitalization rates, length of stay, leaving the hospital against medical advice, readmissions, and in-hospital mortality. When applicable, we separated findings that examined these outcomes among adults living with HCV/HIV coinfection. In addition, we aimed to explore the potential impact of DAAs on these outcomes.

## 2. Methods

**Figure 1:**
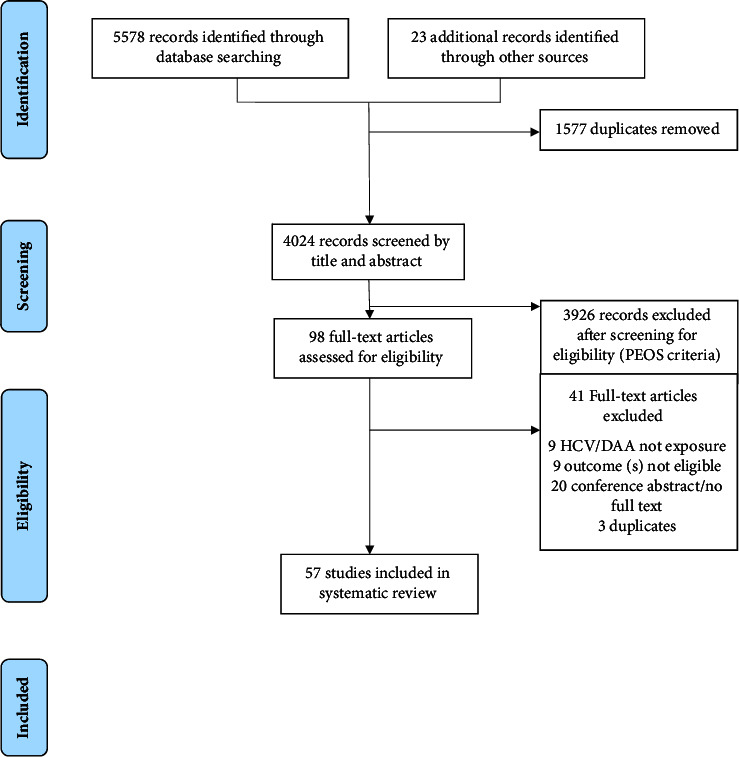
PRISMA 2009 flow diagram.

We referred to the PRISMA and PRISMA-P (Preferred Reporting Items for Systematic Reviews and Meta-Analyses Protocols) Guidelines for the development of this systematic review [17, 18] (see Figure 1 for PRISMA flow diagram). The protocol is also documented in the PROSPERO database (CRD42017081082) [19].

### 2.1. Search Strategy

The search strategy was developed in consultation with a medical reference librarian at the University of British Columbia (U. Ellis, October 5, 2017, personal communication). We searched the following 5 electronic databases to identify relevant studies published from database inception to August 9, 2022: *MEDLINE*, *EMBASE*, *CINAHL*, *PsycINFO*, and *Web of Science*. Search terms were combined using appropriate Boolean operators and included terms relevant to hospital-related outcomes and HCV (see detailed strategy in S1 Table). We mapped the terms to database-specific headings and controlled vocabulary terms when available. We hand-searched reference lists of published literature reviews and included studies.

### 2.2. Eligibility Criteria

The population, exposure, outcomes, and study designs considered in this review are included in Table 1. Only English-language publications were eligible for inclusion, but we did not restrict eligibility to setting or publication date.

### 2.3. Study Selection, Data Extraction, and Synthesis

After the database search and removal of duplicates, two investigators (MN and LA) independently screened titles, abstracts, and full-text articles in three separate stages. At each stage, studies not clearly meeting inclusion criteria were excluded from further review. Disagreements were resolved by consensus, and, if required, discussion with a third investigator (LT). We extracted data using a standardized form, which was then synthesized narratively and in structured tables.

### 2.4. Risk of Bias Assessment

The methodological quality of the included studies was assessed using the Newcastle-Ottawa Scale (NOS) by two investigators (MN and LT) [20]. Studies were evaluated for risk of bias through assessment of selection bias (e.g., representativeness), comparability of groups (e.g., confounding factors), and assessment of outcome(s) (e.g., adequate follow-up).

## 3. Results

### 3.1. Summary of Included Studies

Of the 57 included studies (Figure 1), the majority (*n* = 41) were conducted in North America, while the remaining studies were conducted in Europe (*n* = 9), Asia (*n* = 3), Australia (*n* = 2), Africa (*n* = 1), and spanning multiple continents (*n* = 1). The studies were conducted between 1989 and 2019. The majority of studies employed a retrospective design (*n* = 44), while the remaining studies used a prospective (*n* = 9), cross-sectional (*n* = 3), or combined retrospective and prospective design (*n* = 1). There were 31 unique databases where data were drawn from. Databases that were utilized in more than 1 study included the Nationwide Inpatient Sample (*n* = 6), Medicare (*n* = 5), Healthcare Cost and Utilization Project (*n* = 2), HIV Research Network (*n* = 2), National Hospital Discharge Survey (*n* = 2), Spanish Minimum Basic Data Set (*n* = 2), and the US Renal Data System (*n* = 2). An additional 10 databases were used in 1 included study only. Fourteen studies were conducted among people living with HIV infection. Four available studies examined the impact of DAAs on hospital-related outcomes among people with HCV infection.

### 3.2. Risk of Bias within Studies/Quality Assessment

Most studies relied on anti-HCV serology (*n* = 12), HCV-RNA (*n* = 9), and *International Classification of Diseases*, *Ninth and Tenth Revision*, and *Clinical Modification codes* (*n* = 22) to define their HCV exposure variable. The remaining studies relied on self-report (*n* = 2), were not defined (*n* = 8), or examined DAAs as their exposure variable (*n* = 4). Overall, most studies had good methodological quality with a Newcastle-Ottawa Scale score of at least 6 (*n* = 49). Additional information on study location, design, population characteristics, exposure, outcome (s), and main findings are available in Tables 2 and 3. Study quality scores are available in Table 3.

### 3.3. Hospitalization

There were twenty-six available articles that examined the impact of HCV on hospitalization. Fourteen of these studies analyzed the effect of HCV mono-infection, while twelve studies looked at the effect of HCV coinfection among those living with HIV.

Of the fourteen articles that looked at the effect of HCV mono-infection on hospitalization, twelve studies showed evidence that people living with HCV infection are at an increased odds of hospitalization compared to those without HCV, with odds ratios ranging from 1.09 to 2.74 [23, 28–30, 34, 43, 45, 48, 54, 57, 67, 70]. For example, a retrospective study of commercially insured patients in the US (*n* = 17722) found that in a 1-year duration, those with HCV infection had 15.96% more hospitalizations compared to HCV-negative patients [57]. These findings of a positive association with HCV infection and hospitalization were consistent among other populations, including two US studies of renal and liver transplant recipients (*n* = 7220 and *n* = 28692, respectively) [34, 67] and one international study of patients receiving hemodialysis (*n* = 76689) [23] Similar results were seen in two studies focused on specific hospitalizations for infection and heart failure [30, 54]. The two other studies, which were cross-sectional surveys in Europe and the US, showed no statistically significant difference in annual hospitalization with HCV mono-infection when compared to matched controls [76, 77].

There were twelve studies conducted among people living with HIV infection, of which nine studies showed that HCV coinfection was associated with increased hospitalization, with incidence rate ratios ranging from 1.22 to 1.75 [22, 24, 25, 32, 40, 41, 60, 69, 75]. For example, a large US study of hospitalized patients with HIV (*n* = 263062) found that those with HCV coinfection had an increased hospitalization rate of 23.5 per 100 individuals, compared to 19.9 among those with HIV mono-infection [32]. These findings were consistent among populations with psychiatric illnesses [69]. The three other studies (two conducted in the US and one in Australia) failed to show a statistically significant difference in hospitalization with HCV coinfection [21, 46, 62].

Three studies were available that examined the impact of DAAs on hospitalization, of which all showed evidence that DAAs decrease hospitalization rates [50, 58, 66]. A retrospective study of patients with HCV infection and cirrhosis (*n* = 378) in the US found that DAA treatment was associated with a 64.3% reduction in liver-related hospitalizations [50].

### 3.4. In-Hospital Mortality

Of the thirteen studies that focused on HCV mono-infection and in-hospital mortality, seven of these studies found higher odds of in-hospital mortality among people living with HCV infection compared to those without HCV, with odds ratios ranging from 1.23 to 9.45 [37, 49, 55, 56, 65, 68, 71]. For example, a US study of Medicare patients (*n* = 273132) found that HCV infection increased the odds of in-hospital mortality by 23% [65]. Only one study showed decreased in-hospital mortality among patients with HCV infection. However, this US study of hospitalized patients with viral hepatitis (*n* = 1217165) compared the mortality rates of the HCV-exposed group to HBV as the control group [38]. Another retrospective study in Spain had variable results when examining intensive care unit (ICU) mortality and HCV infection, showing that HCV infection resulted in higher mortality only among those with severe sepsis and compensated cirrhosis, while subgroups without severe sepsis or with decompensated cirrhosis showed no difference in ICU mortality [31]. The four remaining studies showed no difference in in-hospital mortality between patients with and without HCV mono-infection across different populations, including two studies of US patients with alcohol-related diseases [52, 72], one study of Turkish patients undergoing cardiac surgery [33], and one study of patients from a Scottish liver database [70].

There were two studies conducted among people living with HIV infection [32, 59]. A retrospective study of ICU admissions in Spain (*n* = 1891) found increased mortality with HCV coinfection by 44% to 57%, regardless of whether severe sepsis was present [59]. Conversely, a US study of hospitalized patients (*n* = 263062) found no difference in mortality with HCV coinfection [32].

One study was available that examined the impact of DAAs on in-hospital mortality [66]. In this Canadian study of in-patients with chronic HCV and chronic liver disease over the period of 2004 to 2016, DAAs resulted in a 1.9% annual decrease in mortality [66].

### 3.5. Length of Stay (LOS)

Of the ten articles that looked at the effect of HCV mono-infection on LOS, nine studies showed evidence that people living with HCV infection have an increased LOS compared to those without HCV [43, 44, 47, 51, 55, 65, 70–72]. This ranged from an additional 0.85 days to 5.8 days when comparing average LOS. The majority of these studies were conducted in the US. A retrospective study of US Medicare patients (*n* = 273132) found that length of stay was increased by 41.54% among patients with HCV infection [65]. These findings were consistent with various other populations, including two studies among surgical patients [51, 55] and two studies among patients with alcohol-related diseases [71, 72]. In contrast, one study conducted in the US (*n* = 1217165) found that HCV infection decreased LOS by 0.64 days when compared to HBV infection [38].

Among the four studies conducted among people living with HIV infection, the effects of HCV coinfection on LOS were variable when compared to HIV mono-infection [22, 32, 46, 60]. A study of 842 patients from the Australian HIV observation database found that HCV coinfection increased LOS after multivariable analysis [46]. On the other hand, a large US study (*n* = 263062) found that HCV coinfection decreased LOS by 0.4 days [32]. The two other studies, conducted in a US AIDS primary care service and a single Spanish hospital, showed no statistical difference in LOS [22, 60].

### 3.6. Readmission

Of the eleven studies that focused on HCV mono-infection and readmission, nine of these studies found higher odds of readmission among people living with HCV infection compared to those without HCV, with odds ratios ranging from 1.1 to 2.37 [26, 27, 39, 42, 47, 55, 63, 73, 74]. One retrospective study of hospitalized inmates in the US (*n* = 4673) found that HCV infection increased readmission rates at 30 days by 73% [74]. Similar findings were seen among patients with cirrhosis, with two studies conducted in China (*n* = 3402) and the US (*n* = 69612) showing an increased likelihood of readmission with HCV infection [42, 73]. This positive association of HCV infection with readmission was also seen among other populations in North America, such as two studies of surgical patients [39, 55] and two studies of transplant recipients [27, 63]. The two other studies, drawn from a Scottish liver disease database and a US renal database, found no statistically significant difference in readmission with HCV mono-infection [53, 70].

There was one study conducted among people living with HIV infection. This was a retrospective study of inpatients (*n* = 1937) in a Spanish hospital, which found that HCV coinfection increased the risk of 30-day readmission by 10% [60].

One small study examined the impact of DAAs on readmission in a composite endpoint [36]. This single-center US study included 13 kidney transplant recipients with HIV/HCV coinfection and examined the rate of serious infections requiring ICU admission during initial transplant hospitalization or readmission within 6 months. They found that recipients in the DAA era had less serious infections when compared to those in the pre-DAA era (0% versus 67%, *p*=0.02). However, readmissions were not reported separately, therefore limiting conclusions on the specific effect of HCV on this outcome.

### 3.7. Discharge against Medical Advice

There was one study available that included discharge against medical advice as an outcome. This US study of hospitalizations with cirrhosis (*n* = 581380) found that patients with HCV infection as the etiology of liver disease were less likely to self-discharge by 13% when compared to those with non-HCV etiologies [61].

### 3.8. Other Hospital-Related Outcomes

There were three articles available that included in-hospital complications as an outcome, of which all three showed evidence that HCV infection was associated with higher odds of complications during hospitalization, with odds ratios ranging from 1.30 to 2.14 [35, 49, 51]. All three studies were conducted among US patients undergoing elective total joint arthroplasty.

One study examined the impact of HCV infection on medical ICU admissions among patients from the US Veterans Health Administration (*n* = 155550) and showed that HCV infection increased the risk of ICU admissions by 33% [64].

Another study assessed the impact of DAAs on ICU admissions. As described previously, this small US study of kidney transplant recipients with HIV/HCV coinfection found that recipients in the DAA era had lower rates of serious infections requiring ICU admission during initial transplant hospitalization or readmission within 6 months [36]. However, ICU admissions were not reported separately in their data.

## 4. Discussion

### 4.1. Summary of Evidence

Our systematic review found consistently good and high-quality evidence of an association between HCV infection and hospital-related outcomes, primarily increased hospitalizations, length of stay, and readmissions, with a less consistent association with increased in-hospital mortality. To our knowledge, there has been no previous systematic review on HCV infection and hospital outcomes. Our findings demonstrate that chronic HCV infection will continue to impact hospital care as the “baby boomer” cohort advances in age [5, 6, 78]. While there were few studies on DAAs available, those available showed that DAAs were associated with a reduction in hospitalizations and mortality [36, 50, 58, 66]. With the advancement of DAAs, there is growing potential for the widespread treatment of HCV to combat this epidemic and its financial burden on hospital systems [8, 16].

We found that the presence of HCV infection increased hospitalizations, length of stay, and readmissions, including among populations with other comorbidities, such as end-stage renal disease on hemodialysis, post-solid organ transplant, and psychiatric illness. These findings persisted even after the majority of studies had adjusted for a number of confounding variables, including age, sex, race, severity of liver dysfunction, and type and number of comorbidities, including HIV and substance use (e.g., injection drug use and alcohol use). A possible explanation for this may be that HCV infection is often considered a chronic issue and not prioritized by patients and their health-care providers as compared to other pressing issues (e.g., food and housing instability, cancer, cardiovascular disease, and renal disease) [79, 80]. One US study from the pre-DAA era found that many physicians do not address HCV treatment, especially in acute care settings where 64–80% of people diagnosed with HCV did not have a discussion about treatment with their diagnosing physician, compared to 36% diagnosed in the community by a generalist physician [81]. People with chronic HCV infection often have more comorbidities [82–84] and lower socioeconomic status [85], which in themselves are associated with worse hospital outcomes [86–89]. Chronic HCV infection, which is typically asymptomatic, would be less likely to be addressed in people who commonly present with complex medical and psychosocial issues, thereby resulting in the development of severe metabolic complications and negative hospital outcomes later on.

In the fourteen studies conducted among people living with HIV, we found that those with HCV coinfection similarly and consistently had increased hospitalizations. However, unlike our findings among those without HIV, there was less evidence of a consistent relationship for length of stay, in-hospital mortality, and readmissions. One possible explanation may be that differences in medical and psychosocial factors between those with and without HIV coinfection may affect their hospital visits [90–92]. In particular, given the increased effort to deliver HIV treatment globally [93], those with HIV coinfection may be more likely to be connected to health care compared to those without HIV infection. Multiple studies in North America have shown that HIV coinfection and being connected to health care such as through antiretroviral therapy or opioid agonist treatment were associated with higher treatment uptake for HCV [94–98]. Therefore, while HCV coinfection still led to increased hospitalizations among those living with HIV, regular engagement in health care may mitigate and be protective in regard to the other negative hospital outcomes examined in this systematic review.

There was very limited evidence that discussed the impact of DAAs on hospital outcomes, with only three studies found [50, 58, 66]. While the studies available showed that DAAs were associated with fewer hospitalizations and in-hospital mortality, this is an identified gap in knowledge. With the *World Health Organization's* global targets for 2030 of having 80% of people on treatment, 90% of new infections reduced, and 65% reduction in mortality, it is clear that DAAs will play a prominent role in the global HCV strategy [2, 99]. There is a need for future studies to better understand the effects of DAAs on short- and long-term hospital-related outcomes and the reduction of associated costs. The majority of studies examining hospitalization did not describe the reason for admission (*n* = 23), and therefore, we are unable to conclude whether HCV cure with DAAs would significantly impact hospital outcomes in people with HCV as well as other comorbidities. Nevertheless, previous studies have consistently shown the benefits of DAAs for curing HCV, including among the most marginalized populations such as people who inject drugs [13, 95, 100]. Therefore, efforts to scale up access and uptake of these medications through interventions that account for individual, social, structural, and environmental influences are critical for ensuring equitable access.

## 5. Limitations

Several limitations should be considered when interpreting our findings. First, as most studies in our review were observational, we are unable to infer a causal association between HCV and negative hospital outcomes. While many of the studies reviewed used multivariable analyses, it is possible that our findings are due to unmeasured confounding. However, this is a common limitation of observational studies that is unlikely to be overcome, given the unethical nature of randomizing people to HCV infection. Furthermore, the study designs for most included studies were retrospective in nature, which could lead to biased results. Second, the variation in the definition of HCV infection limits our ability to make confident interpretations. While 21 studies used anti-HCV serology or HCV-RNA levels to define HCV infection as the exposure variable, the majority of studies relied on *ICD* codes or were undefined, causing uncertainty as to whether HCV infection was appropriately confirmed, or whether it was an acute or chronic infection. Third, the majority of studies were completed in high-income countries, with a significant portion among people with HIV coinfection, which may affect the generalizability of our results. Fourth, as with any review of literature, our findings are subject to publication bias, which may weigh results towards significance. Lastly, it is possible that we may have missed some pertinent literature in our search strategy, particularly non-English studies and conference abstracts. While screening and selection of studies are subjective, we attempted to minimize this bias by using two independent reviewers during the screening process.

## 6. Conclusions and Future Directions

In summary, this systematic review found good to high-quality evidence that HCV negatively impacts hospital outcomes, primarily through increased hospitalizations, length of stay, and hospital readmissions. There was a paucity of studies on the effect of DAAs on hospital outcomes. As DAAs become more available and accessible, future research is needed to understand their impact on hospital-related outcomes. [101].

## Figures and Tables

**Table 1:** Population, exposure, outcomes, and study design criteria (PEOS) for study inclusion.

Criteria	Definition/description
Population	≥18-year-old adults at baseline
Exposure	Acute or chronic HCV infection; direct-acting antivirals
Outcomes	Hospital-related outcomes (hospitalization, length of stay, leaving hospital against medical advice, readmission, and in-hospital mortality)
Design	Original quantitative research studies (including observational studies and randomized controlled trials)^†^

†Commentaries, letters to the editors, editorials, and other types of opinion pieces were excluded. Literature reviews were excluded, but back referencing was conducted to ensure that all relevant studies from the literature review were included.

**Table 2:** Summary of included studies.

Author (year)	Study period/study location	Database/study	N/population	Age/% male/ethnicity	HIV positive (%)	Substance use (%)
*Prospective cohort studies*						
Gardner et al., 2003 [21]	1993–2000/US	HIV Epidemiology Research Study	*N* = 885/women aged 16–55 years with HIV infection	Age: ≤30 years 21.4%, 31–44 years 68.4%/male 0%/African American 60.8%, White 20.7%, Hispanic 17.2%, and other 1.4%	100%	60.7%
Gebo et al., 2003 [22]	1995–2000/US	—	*N* = 3730/patients with HIV infection	Mean age: 37.2 years (range 17–82)/male 67.5%/White 21.5%, African American 76.9%, and other 1.5%	100%	47.4%
Goodkin et al., 2017 [23]	1996–2015/Australia, Belgium, Canada, China, France, Germany, Bahrain, Kuwait, Oman, Qatar, Saudi Arabia, United Arab Emirates, Italy, Japan, New Zealand, Spain, Russia, Sweden, Turkey, UK, and US	Dialysis Outcomes and Practice Patterns Study	*N* = 76689/age ≥18 years on hemodialysis	Mean age: 62.5 years/male 58.7%/Black 28.4%†	0.8%	2% in the last 12 months
Linas et al., 2011 [24]	2000–2007/US	ACTG Longitudinal Linked Randomized Trials (ALLRT)	*N* = 3143/patients with HIV infection	Mean age: 40 years (SD 9.2)/male 83.3%/White 50.2%, African American 27.8%, Hispanic 19.2%, and other 2.7%	100%	9.7%
Mena et al., 2017 [25] ‡	1993–2014/Spain	--(University Hospital of A Coruna, Spain)	*N* = 2379/people with HIV infection	Mean age: 32.2 years at HIV diagnosis/male 75.2%/ethnicity NR	100%	48.8%
Rezk & Omar, 2017 [26]	2012–2017/Egypt	—	*N* = 512/pregnant women	Age: 20–30 years 61.7%, >30 years 38.3%/male 0%/ethnicity NR	NR	0%
Shankar et al., 2011 [27]	2007–2011/Canada	—	*N* = 208/age ≥18 years undergoing primary liver transplantation	Mean age: 53.1 years (SD 9.8)/male 78.8%/ethnicity NR	NR	NR
Tandon et al., 2015 [28]	2001–2011/US	OptumHealth Reporting and Insights Database	*N* = 19682/age ≥18 years with private insurance	Mean age: 52.0 years/male 61.4%/ethnicity NR	Excluded	4.0%
Teshale et al., 2016 [29]	2006–2013/US	Chronic Hepatitis Cohort Study	*N* = 30393/age ≥18 years	Age: 46–60 years 69.2%/male 59.2%/White 63.1%, Black 23.7%, Hispanic 3.9%, Asian 3.3%, Hawaiian/Pacific Islander 1.4%, American Indian/Alaska Native 1.4%, and unknown 3.2%	Excluded	NR
Tsui et al., 2009 [30]	2000–2002/US	Heart and Soul Study	*N* = 981/outpatients with coronary heart disease	Mean age: 66.3 years/male 81.9%/non-White 39.7%	2.5%	7.6%

*Retrospective cohort studies*						
Alvaro-Meca et al., 2016 [31]	2005–2010/Spain	Spanish minimum basic data set	*N* = 5265/age ≥19 years with cirrhosis admitted to ICU	Median age: chronic hepatitis C 58.2 years, control 57.2/male 65.6%/ethnicity NR	Excluded	37.3% alcohol and drug abuse
Ananthakrishnan et al., 2010 [32] §	2006/US	Nationwide inpatient sample	*N* = 263062/hospitalized patients with HIV and/or HCV infection	Age for coinfected group: 36–50 years 55.7%, 51–65 years 33.3%/male 63.4%/White 38.2%, Black 26.6%, Hispanic 13.0%, other 3.2%, and missing 19.0%	35.6%	NR
Baran et al., 2018 [33]	2005–2016/Turkey	TurcoSCORE database	*N* = 120/patients undergoing cardiac surgery	Mean age: 61.1 years/male 50.8%/ethnicity NR	NR	NR
Batty et al., 2001 [34]	1994–1997/US	US renal data system	*N* = 28692/renal transplant recipients	Mean age: 43.0 years/male 60.4%/ethnicity inconsistent data	NR	Inconsistent data
Best et al., 2015 [35]	1990–2007/US	National hospital discharge survey	*N* = 8363266/noncirrhotic patients admitted for primary THA or TKA	Mean age: 67.3 years/male 38.5%/ethnicity NR	Excluded	NR
Camargo et al., 2019 [36]	2007–2017/US	—	*N* = 13 (7 DAA, 6 pre-DAA)/Coinfected HIV/HCV kidney transplant recipients	Median age: 56 years (IQR 47–63)/male 84.6%/African American 76.9%	100%	NR
Chen et al., 2002 [37]	1992–1998/Taiwan	—	*N* = 252/patients who underwent hepatic resection for hepatocellular carcinoma	Mean age: 54.3 years (range 19–87)/male 77.8%/ethnicity NR	NR	NR
Cholankeril et al., 2016 [38]	2003–2012/US	Nationwide inpatient sample	*N* = 1217165/adults hospitalized with HCV or HBV	Mean age: 51.4 years/male 62.3%/White 48.4%, Black 4.8%, Hispanic 11.4%, Asian 2.3%, and other 3.4%	AIDS 3.6%	23.4%
Chowdhury et al., 2017 [39]	2006–2014/US	Tricare insurance claims (from Military Data Repository)	*N* = 2262/age 18–64 years who received major orthopaedic surgery (spine, THA, and TKA)	Mean age: 52.6 years (SD 8.7)/male 51.5%/White 51.7%	NR	NR
Crowell et al., 2014 [40]	2010/US	HIV research network	*N* = 12819/adults with HIV infection	Median age: 47 years (IQR 40–53)/male 71.7%/White 26.7%, Black 49.2%, Hispanic 20.8%, and other/unknown 3.4%	100%	17.4%
Crowell et al., 2015 [41]	2006–2011/US	HIV research network	*N* = 15927/adults with HIV infection	Median age: HIV mono-infection 40.4 years (IQR 32.6–47.0), HIV/HCV coinfection 47.0 years (42.0–51.9)/male 76.8%/White 35.7%, Black 43.5%, Hispanic 17.7%, and other/unknown 3.1%	100%	16.7%
Dai et al., 2019 [42]	2012–2015/China	—	*N* = 3402/age ≥18 years admitted with cirrhosis	Age: 40–59 years 60.4%, ≥60 years 21.6%/male 71.3%/Han 73.6%, and minority 26.4%	Excluded	NR
Davis et al., 2011 [43]	2002–2006/US	Integrated Health Care Information Services (IHCIS) Managed Care Benchmark Database	*N* = 41324/patients enrolled in the US claims database with no HBV diagnoses, ≥6 and ≥12 months of continuous plan enrollment pre- and postdiagnosis of chronic HCV	Mean age: 48.9 years/male 60.7%/ethnicity NR	NR	NR
Deshpande et al., 2019 [44]	2005–2016/US	Medicare	*N* = 291663/patients with HCV infection receiving hemodialysis	Mean age: 67.3 years (SD 15.2)/male 55.0%/White 55.0%, Black 32.5%, Asian 3.0%, Hispanic 5.8%, and Native American 1.4%	NR	NR
Duberg et al., 2011 [45]	1990–2006/Sweden	Swedish Institute for Infectious Disease Control—National Surveillance Database	*N* = 258000/individuals diagnosed with HCV infection, compared to the general population	Mean age: 38.3 years (SD 12.8)/male 69.1%/Country of origin: Nordic 89.3%, non-Nordic European 4.9%, Asia 3.4%, Africa 1.1%, South America 0.8%, and North America 0.4%	NR	9.6% in HCV group
Falster et al., 2010 [46]	1999–2007/Australia	Australian HIV Observational Database	*N* = 842/patients with HIV infection	Median age: 41 years (IQR 35–48)/male 94.4%/ethnicity NR	100%	NR
Fukui et al., 2017 [47]	2010–2014/US	Medicare	*N* = 37165/hospitalized patients age ≥65 years or with qualified health conditions discharged to hospice	Mean age: 82.3 years/male 40.4%/White 87.2%, Black 8.3%	NR	NR
Gidding et al., 2010 [48]	1992–2006/Australia	New South Wales Notifiable Diseases Database	*N* = 82601 (HCV only)/people notified of HCV mono-infection, compared to the general population of New South Wales	For the HCV group only,median age 37 years (IQR 29–44)/male 63.0%/ethnicity NR	Excluded	Hospitalization rate 29.0/1000 person-years for illicit drug use
Grau et al., 2018 [49]	1990–2007/US	National Hospital Discharge Survey	*N* = 4717536/noncirrhotic patients hospitalized with surgically treated hip fractures	Mean age: 79 years/male 26.1%/ethnicity NR	Excluded	NR
Hill et al., 2018 [50]	2011–2017/US	—	*N* = 378 (196 DAA, 182 untreated)/age ≥18 years with chronic active HCV with cirrhosis	Median age: DAA 59 years (range 18–82), untreated 56 years (range 35–74)/male 61.1%/White 56.3%, Black 11.4%, Asian 3.4%, other 26.7%, and unknown 2.1%	6.3%	NR
Issa et al., 2015 [51] #	1998–2010/US	Nationwide Inpatient Sample	*N* = 25372/patients who underwent primary THA or TKA	Mean age: 66.4 years/male 39.0%/White 86%, and non-White 14%	NR	NR
Kim et al., 2001 [52]	1995/US	Healthcare Cost and Utilization Project Database	*N* = 283000/hospitalizations related to liver disease from HCV infection or alcohol	Median age: HCV/ETOH 43 years, ALD 51 years/male 69.6%/ethnicity NR	3.1%	NR
King et al., 2016 [53]	1999–2011/US	US Renal Data System, Medicare	*N* = 3643/adult primary first-time simultaneous pancreas-kidney recipients	Mean age: 40.1 years/male 64.2%/African American 20.6%	NR	NR
Lee et al., 2019 [54]	2005–2008/Taiwan	Taiwan National Health Insurance Research Database	*N* = 115336/age ≥20 years without cirrhosis participating in the New Taipei City Health Screening	Mean age: 52.2 years/male 35.6%/ethnicity NR	0%	0%
Mahure et al., 2018 [55]	2010–2014/US	New York Statewide Planning and Research Cooperative System (SPARCS) Database	*N* = 80722/age ≥18 years undergoing elective inpatient THA	Mean age: 65 years/male 44.7%/White 80.7%, Black 7.8%, Hispanic 3.8%, and other 7.7%	0.8%	1.1%
Marrie et al., 2017 [56]	2000–2014/Canada	Provincial Lab for Public Health (Edmonton, Alberta)	*N* = 3251/age ≥17 years with invasive pneumococcal disease	Mean age: 54.6 years/male 56.5%/aboriginal 13.8%	4.2%	17.2%
McCombs et al., 2011 [57]	2003–2008/US	Unknown Health Insurance Company	*N* = 17722/commercially insured patients	Mean age: 49.6 years/male 60.1%/ethnicity NR	Excluded	NR
McDonald et al., 2019 [58]	2013–2018/Scotland	HCV Clinical Database	*N* = 1073/patients with chronic HCV infection and compensated cirrhosis, initiated on IFN-free DAA	Age: 45–54 years 42.4%/male 74.6%/ethnicity NR	NR	62.6% PWID
Medrano et al., 2014 [59]	2005–2010/Spain	Spanish Minimum Basic Data Set	*N* = 1891/age ≥19 years with HIV infection admitted to ICU	Median age: 43 years/male 77.5%/ethnicity NR	100%	49.8% alcohol and drug abuse
Meijide et al., 2017 [60]	1993–2013/Spain	-University Hospital of A Coruna, Spain	*N* = 1937/hospitalized patients with HIV infection	Mean age: 36.4 years (SD 10.6)/male 75.0%/ethnicity NR	100%	NR
Myers et al., 2009 [61]	1993–2005/US	Nationwide Inpatient Sample	*N* = 581380 hospitalizations/age ≥18 years hospitalized with cirrhosis	Mean age: 57.0 years/male 60.8%/White 54%, African American 8.7%, Hispanic 12.4%, Asian/Pacific Islander 1.4%, and other 23.5%	0.9%	5.0%
Norton et al., 2012 [62]	1996–2010/US	—	*N* = 261/age ≥18 years with HIV-1 mono-infection	Median age: 50 years/male 75.9%/White 40.6%, Black 54.4%, and other 5.0%	100%	18.0%
Patel et al., 2016 [63]	2005–2015/US	UNOS Standard Transplant Analysis and Research Registry	*N* = 325/age ≥19 years who underwent deceased donor liver transplantation	Mean age: 55.9 years (SD 8.8)/male 78.7%/Black 7.1%, Hispanic 4.6%, other 4.0%, and White 84.3%	NR	NR
Rentsch et al., 2019 [64]	1997–2014/US	Veterans Aging Cohort Study (Veterans Health Administration), Medicare	*N* = 155550/patients in the veterans health administration with and without HIV, HCV, and alcohol-related diagnoses	Median age: 47 years (IQR 40–54)/male 97.3%/Black 47.1%, White 39.9%, Hispanic 8.2%, and other/unknown 4.8%	32.0%	13.8%
Sayiner et al., 2016 [65]	2005–2010/US	Medicare	*N* = 273132/patients born between 1945 and 1965	Age: 50–54 years 25.5%, 55–59 years 30.5%/male 50.2%/White 72.3%, and non-White 27.7%	NR	NR
Schanzer et al., 2018 [66]	2004–2016/Canada	Canadian Discharge Abstract Database	*N* = 2115–3255 hospitalizations per year, 331–464 inpatient deaths per year/inpatients diagnosed with chronic HCV and chronic liver disease	NR	NR	NR
Sharma et al., 2017 [67]	2003–2010/US	Scientific Registry of Transplant Recipients	*N* = 7220/age ≥18 years and older who underwent deceased donor liver transplantation	Median age: 59 years (IQR 52–66)/male 66.4%/White 73.9%, Black 7.6%, Asian 3.8%, Hispanic/Latino 13.6%, multiracial/other 1.1%	NR	NR
Singal et al., 2012 [68]	1998–2007/US	Nationwide Inpatient Sample	*N* = 111726/patients hospitalized with alcoholic hepatitis	Age: 40–50 years 36.3%, 50–60 years 25.9%/male72.8%/Hispanic 7.8%, caucasian 52.6%, African American 11.0%, and others 28.7%	NR	NR
St-Jean et al., 2019 [69]	2000–2015/Canada	BC Seek and Treat for Optimal Prevention of HIV/AIDS	*N* = 4046/age ≥18 years with HIV infection and ART-naïve, initiating treatment	Age: 30–39 years 30.5%, 40–49 years 35.4%/male 81.9%/ethnicity NR	100%	34.4%
Steinke et al., 2002 [70]	1989–1999/Scotland	Epidemiology of Liver Disease in Tayside (ELDIT) Database	*N* = 1407 (with matched control), 366849 (general population)/registrants in a liver disease database, compared to general population in tayside, scotland	Mean age: HCV 34.8 years (SD 15.4), general population 42.5 years (SD 23.1)/male: HCV 68.4%, general population 48.9%/ethnicity NR	NR	OR 50.5 (95% CI 16.1–159.1) for methadone treatment in HCV group
Thuluvath et al., 2013 [71]	1998–2006/US	Nationwide inpatient sample	*N* = 112351/noncirrhotic patients hospitalized with alcoholic hepatitis	Mean age: 46.5 years/male 67.5%/ethnicity NR	NR	NR
Tsui et al., 2006 [72]	1996–2002/US	—	*N* = 6532/patients hospitalized with alcohol dependence or abuse	Mean age: 46.8 years/male 80.7%/White 41.0%, Black 33.1%, Hispanic 18.1%, Asian 3.2%, and unknown/other 4.7%	NR	NR
Wei et al., 2018 [73]	2009–2011/US	Healthcare Cost and Utilization Project State Inpatient Database, California	*N* = 69612/age ≥18 years hospitalized with cirrhosis	Mean age: 59.4 years/male 61.9%/Caucasian 69.4%, African American 6.9%, Hispanic 0.7%, Asian/Pacific Islander 6.4%, Native American 15.8%, and other 0.7%	NR	NR
Wurcel et al., 2018 [74]	2004–2014/US	—	*N* = 4673/hospitalized inmates age ≥18 years	Median age: 44 years (IQR 34–52)/male 92.6%/White 53.3%, Black 21.2%, Hispanic 12.0%, other 13.5%, missing data 1.5%	2.5%	NR

*Cross-sectional studies*						
Baum et al., 2008 [75]	2002–2006/US	—	*N* = 192/age ≥19 years with HIV infection and active drug use	Mean age: 42.3 years/male 74.5%/African American 78.2%, Hispanic 15.1%, White non-Hispanic 6.3%, others 0.5%	100%	100%
El Khoury et al., 2012 [76]	2010/US	US National Health and Wellness Survey	*N* = 612/age ≥18 years	Mean age: 53.4 years/male 62.1%/White 72.9%, Black 12.1%, Hispanic 7.5%, and other 7.5%	Excluded	NR
Vietri et al., 2013 [77]	2010/France, Germany, UK, Italy, Spain	European National Health and Wellness Survey	*N* = 572/age ≥18 years	For HCV group only, mean age: 52.8 years (SD 13.49)/male 58.4%/ethnicity NR	Excluded	NR

AIDS: acquired immunodeficiency syndrome; ALD: alcohol-induced liver disease; ART: antiretroviral therapy; CI: confidence interval; DAA: direct-acting antiviral; HBV: hepatitis B virus; HCV: hepatitis C virus; HCV/ETOH: concurrent hepatitis c and alcohol abuse or alcohol-induced liver disease; HIV: human immunodeficiency virus; ICU: intensive care unit; IFN: interferon; IQR: interquartile range; NR: not reported; OR: odds ratio; PWID: people who inject drugs; SD: standard deviation; THA: total hip arthroplasty; TKA: total knee arthroplasty; US: United States; UK: United Kingdom. †Ethnicity data only available from North America, which made up 38.4% of total *N*. ‡Prospective and retrospective cohort study. §Analysis based on *N* = 263062 (HIV/HCV coinfection 56304; HIV mono-infection 206758). Demographic information based on *N* = 737905 including coinfection, HIV mono-infection and HCV mono-infection. ¶Data reported for ethnicity and substance use was inconsistent between percentage reported and absolute numbers reported. #Demographic information (age, sex, and ethnicity) based on *N* = 1700400 with HCV 0.47%.

**Table 3:** Main findings and assessment of methodological quality of included studies.

Author (year)	HCV-positive (%)	Main exposure	Measurement	Study outcome/main findings	Risk of bias (Newcastle Ottawa Scale)
Gardner et al., 2003 [21]	61.4%	HIV/HCV coinfection	Anti-HCV serology	Hospitalization: ARR 1.0 (0.8–1.2)	6
Gebo et al., 2003 [22]	42.8%	HIV/HCV coinfection	Anti-HCV serology	Mean LOS: 7.0 (range 0–71) vs. 7.1 (range 0–150) days, *p*=0.52	7
				Hospitalization: AIRR 1.75 (1.47–2.07)	
Goodkin et al., 2017 [23]	7.5%	HCV	Established diagnosis or positive serology	Hospitalization: AOR 1.09 (1.04–1.13)	6
Linas et al., 2011 [24]	11.8%	HIV/HCV coinfection	Anti-HCV serology	Hospital nights: adjusted relative risk 1.8 (1.3–2.5)	5
Mena et al., 2017 [25] †	41.6% (35% chronic HCV/HIV and 6.1% HCV spontaneous resolvers)	HIV/HCV coinfection spontaneous resolvers and HIV/chronic HCV infection	Anti-HCV serology and HCV-RNA	Liver-related hospitalization:	6
				(i) Chronic HCV/HIV ASHR 6.92 (*p* < 0.001)	
				(ii) HCV spontaneous resolvers ASHR 1.35 (NSS)	
Rezk & Omar, 2017 [26]	66.8% (35.9% HCV-RNA-, 30.9% HCV-RNA+)	HCV	Anti-HCV serology and HCV-RNA	Repeated hospitalizations (>2): 18 events (HCV-RNA-) and 28 (HCV-RNA+) vs. 4 (control), chi-square 21.9, *p* < 0.001	5
Shankar et al., 2011 [27]	25.5%	HCV as etiology of liver disease	Not defined	Readmission within 90 days: AHR 1.91 (1.17–3.14)	5
Tandon et al., 2015 [28]	50.0%	Chronic HCV	2+ claims with ICD-9-CM code)	Hospitalizations: RR 2.45 (2.37–2.54)	8
Teshale et al., 2016 [29]	33.3%	Chronic HCV	Not defined	Hospitalizations: 27.4 (27.0–27.8) vs. 7.4 (7.2–7.5)/100 patient-years	7
Tsui et al., 2009 [30]	8.6%	HCV	Anti-HCV serology	Heart failure hospitalization: AHR 2.13 (1.19–3.80)	9

*Retrospective cohort studies*					
Alvaro-Meca et al., 2016 [31]	21.6%	Chronic HCV	ICD-9-CM code	ICU mortality stratified by severe sepsis (SS) and compensated cirrhosis (CC)/decompensated cirrhosis (DC):	8
				(i) SS/CC AHR 1.35 (1.11–1.65)	
				(ii) Non-SS/CC AHR 1.10 (0.93–1.31)	
				(iii) SS/DC AHR 1.09 (0.96–1.25)	
				(iv) Non-SS/DC AHR 1.10 (0.97–1.21)	
Ananthakrishnan et al., 2010 [32]^‡^	72.0% (7.6% coinfected, 64.4% HCV mono-infection)	HIV/HCV coinfection	ICD-9-CM code	In-hospital mortality: AOR 1.11 (0.97–1.29)	8
				ALOS: −0.4 days (−0.58 to −0.14)	
				Hospitalization: 23.5 vs. 19.9/100 individuals, *p* < 0.0001	
Baran et al., 2018 [33]	50.0%	Chronic HCV	Anti-HCV serology, HCV-RNA <25 IU/ml	In-hospital mortality: 5% vs. 1.7%, *p*=0.61	6
Batty et al., 2001 [34] §	5.7%	HCV as the dependent variable	Not defined	Hospitalization: associated with HCV AOR 1.28 (1.14–1.45)	6
Best et al., 2015 [35]	0.3%	HCV	ICD-9-CM code	In-hospital complication: AOR 1.686 (1.645–1.727)	7
Camargo et al., 2019 [36]	100%	Post-DAA treatment, compared to pre-DAA on PI-containing ART	—	Serious infections requiring ICU admission during initial transplant hospitalization or readmission within 6 months: 0% vs. 67%, *p*=0.02	4
Chen et al., 2002 [37]	35.3% (HCV 26.2% and HBV/HCV 9.1%)	HCV ± HBV	Anti-HCV serology, HbsAg	In-hospital mortality:	6
				HBV+/HCV+ 17.4% and HBV-/HCV+ 12.1% vs	
				HBV+/HCV- 3.8% and HBV-/HCV- 3.3% *p*=0.021	
Cholankeril et al., 2016 [38]	88.2%^b^	HCV, compared to HBV^c^	ICD-9-CM code	In-patient mortality: AOR 0.74 (0.72–0.77)	8
				Mean ALOS: −0.64 days (−0.69 to −0.61)	
Chowdhury et al., 2017 [39]	50.0%	HCV	ICD-9 code	Readmission at 30 days: OR 1.46 (1.04–2.05)	7
				Readmission at 90 days: OR 1.29 (1.00–1.67)	
Crowell et al., 2014 [40]	17.9% (15.4% HIV/HCV and 2.5% HIV/HBV/HCV)	HIV/HCV coinfection	Anti-HCV serology	Hospitalization: AIRR 1.45 (1.21–1.74)	6
Crowell et al., 2015 [41]	13.6% (12.9% HIV/HCV and 0.7% HIV/HBV/HCV)	HIV/HCV coinfection	Anti-HCV serology	Inpatient visits: AIRR 1.22 (1.10–1.36)	6
Dai et al., 2019 [42]	12.5%	HCV, compared to HBV	Medical records	Readmission within 1 year: AOR 1.51 (1.19–1.91)	8
Davis et al., 2011 [43]	50.0%	Chronic HCV	ICD-9-CM code	In 12 month follow-up period	7
				(i) Mean # hospitalizations: +0.37, *p* < 0.0001	
				(ii) Mean # inpatient days (for those with at least 1 hospitalization): +3.01, *p* < 0.0001	
Deshpande et al., 2019 [44]	4.2%	HCV	ICD-9 and ICD-10 codes)	Mean LOS:	6
				27.9 ± 30.5 days vs	
				22.1 ± 28.1 days per patient per year, *p* < 0.0001	
Duberg et al., 2011 [45]	16.7%	HCV	Anti-HCV serology or HCV-RNA	Hospitalization:	8
				(i) HR 4.03 (95% CI 3.98–4.08)	
				(ii) ARR 5.91 (95% CI 5.87–5.94)	
				Hospital days: ARR 8.78 (95% CI 8.76–8.80)	
Falster et al., 2010 [46]	11.5%	HIV/HCV coinfection	Anti-HCV serology	Hospitalizations: IRR 0.78 (95% CI 0.53–1.16)	5
				Increased LOS: multivariate *β* (SE) = 0.32 (0.14), *p*=0.023	
Fukui et al., 2017 [47]	1.5%	HCV	ICD-9 code	Total annual hospital ALOS: 25.9% higher, *p* < 0.001	7
				Readmission within 30 days: AOR 2.18 (95% CI 1.8–2.6)	
Gidding et al., 2010 [48]	1.2% of NSW hospitalizations in HCV mono-infected	HCV, compared to the general population	Not defined	Hospitalization: 42% higher than the general population, SHR 1.4 (95% CI 1.4-1.4)	8
Grau et al., 2018 [49]	0.1%	HCV	ICD-9 code	In-hospital complication: AOR 2.143 (95% CI 2.024–2.268)	7
				Nonroutine discharge (discharge to another inpatient facility or inpatient mortality): AOR 3.559 (95% CI 3.354–3.776)	
Hill et al., 2018 [50]	100%	DAA treatment, compared to untreated	—	Liver-related hospitalization:	9
				(i) 64.3% reduction or −18.7 (95% CI -11.5 to −25.9) hospitalizations per 100 person-years	
				(ii) No DAA therapy had AOR 3.05 (95% CI 1.68–5.64) for liver-related hospitalization	
Issa et al., 2015 [51]	25.0%^d^	HCV	ICD-9 code	In-hospital complication: OR 1.30 (95% CI 1.17–1.44)	7
				Mean LOS: 13.53% longer (95% CI 12.26-14.82)	
Kim et al., 2001 [52]	22.9%	Concurrent alcohol abuse or ALD and HCV	ICD-9-CM code	In-hospital mortality: AOR 1.0 (95% CI 0.9–1.1)	7
King et al., 2016 [53]	3.7%	HCV	Not defined	Early hospital readmission within 30 days: NSS in preliminary model, excluded from published final model	6
Lee et al., 2019 [54]	2.5%	HCV mono-infection	Anti-HCV serology	Hospitalization for infection: AHR 1.22 (95% CI 1.12–1.33)	9
Mahure et al., 2018 [55]	0.8%	HCV mono-infection	ICD-9 code	In-hospital mortality: AOR 9.45 (95% CI 8.84–11.65)	7
				LOS: AOR 1.32 (95% CI 1.22–1.64)	
				Extended LOS (>90^th^ percentile of all patients): AOR 2.05 (95% CI 1.60–2.37)	
				Readmission within 90 days: AOR 1.90 (95% CI 1.45–2.64)	
Marrie et al., 2017 [56]	10.9%	HCV	Anti-HCV serology documented in chart	In-hospital mortality: AOR 1.71 (95% CI 1.15–2.54)	8
McCombs et al., 2011 [57]	50.0%	HCV	2+ claims with ICD-9 code or 1+ claim HCV drug	In 1 year postindex date	8
				(i) % Hospitalized: +15.96%, *p* < 0.0001	
				(ii) Hospitalizations: AOR 2.620, *p* < 0.0001	
McDonald et al., 2019 [58]	100%	DAA treatment status: nonresponder and noncompliant, on treatment compared to responder	HCV-RNA at the end of treatment and 12 weeks posttreatment	1st decompensated cirrhosis hospitalization:	7
				(i) On-responder AHR 6.90 (95% CI 2.59-18.4)	
				(ii) Noncompliant AHR 2.12 (95% CI 0.97–6.65)	
				(iii) On treatment AHR 1.38 (95% CI 0.27–7.07)	
				1st HCC hospitalization:	
				(i) Nonresponder AHR 5.73 (95% CI 1.26-26.1)	
				(ii) Noncompliant AHR 2.94 (95% CI 0.65-13.3)	
				(iii) On treatment AHR 0.99 (95% CI 0.11–8.76)	
Medrano et al., 2014 [59]	37.0%	HIV/HCV coinfection	ICD-9-CM code	ICU mortality stratified by severe sepsis (SS):	8
				(i) SS AHR 1.44 (95% CI 1.30–1.59)	
				(ii) Non-SS AHR 1.57 (95% CI 1.38–1.78)	
Meijide et al., 2017 [60]	37.2%	HIV/HCV coinfection	HCV-RNA	Hospitalization:	6
				(i) Median # per patient 3.0 (range 1.0–6.0) vs. 2.0 (range 1.0–3.0), *p* < 0.001	
				(ii) Median total days of hospitalization 36.0 days (range 14.0–77.5) vs. 23.0 days (9.0–51.0), *p* < 0.001	
				Readmission within 30 days: relative risk 1.1 (95% CI 1.0–1.2)	
				LOS: median 10.1 days (range 6.3–15.9) vs. 11.0 (range 6.0–19.5), *p*=0.24	
Myers et al., 2009 [61]	20.5%	HCV as etiology of liver disease	ICD-9-CM code	Self-discharge: AOR 0.87 (95% CI 0.82–0.91)	9
Norton et al., 2012 [62]	36.8%	HIV/chronic HCV coinfection	Anti-HCV serology, HCV-RNA positive for greater than 6 months	Hospitalizations: adjusted risk ratio 1.24 (95% CI 0.73–2.09)	8
Patel et al., 2016 [63]	49.9%	HCV is the primary cause of liver failure, compared to alcohol	Not defined	Readmission within 90 days: AOR 2.37 (95% CI 1.44–3.91)	7
Rentsch et al., 2019 [64]	13.0% (2.4% mono-infection)	HCV mono-infection	HCV-RNA	Medical ICU admission: ARR 1.33 (95% CI 1.27–1.39)	9
Sayiner et al., 2016 [65]	5.1%	HCV	ICD-9-CM code	In-hospital mortality: AOR 1.23 (95% CI 1.16–1.29)	9
				ALOS: increased 41.54% (95% CI 39.11–44.01)	
Schanzer et al., 2018 [66]	100%	DAA (2012–2017), compared to pre-DAA (2004–2011)	—	Hospitalization: 32% reduction (95% CI 27%–37%) in 2016/17 compared to pre-DAA baseline projection	8
				In-hospital mortality: AAAPC -1.9% (95% CI -2.6% to −1.1%) from 2003 to 2016	
Sharma et al., 2017 [67]	35.7%	HCV	Not defined	Early hospitalization (within first 6 months of liver transplant): ARR 1.12 (95% CI 1.03–1.21)	7
Singal et al., 2012 [68]	6.5%	HCV	ICD-9 code	In-hospital mortality: AOR 1.29 (95% CI 1.12–1.49)	8
St-Jean et al., 2019 [69]	36.1%	HIV/HCV coinfection ± mental health disorder	Anti-HCV serology or HCV-RNA or physician report	Hospitalization:	7
				(i) Without MHD ARR 2.01 (95% CI 1.71–2.36)	
				(ii) With MHD ARR 2.53 (95% CI 2.20–2.92)	
Steinke et al., 2002 [70]	33.3%	HCV	Anti-HCV serology	Median LOS: 3 days (range 1–138) vs. 2 days (range 1–132), *p* < 0.05	7
				Mean LOS per hospital stay: 5.46 vs. 4.61 days, *p* > 0.05	
				Hospitalization: OR 2.74 (95% CI 2.10–3.58)	
				Mean # hospitalizations: 4.7 vs. 1.5, *p* > 0.05	
				Readmissions per patient: 5.0 vs. 1.6, *p* > 0.05	
				In-hospital mortality: 15 vs. 8, *p* > 0.05	
Thuluvath et al., 2013 [71]	6.1%	HCV	ICD-9-CM code	In-hospital mortality: AOR 1.48 (95% CI 1.10–1.98)	8
				ALOS: increased ratio 1.10 (95% CI 1.05–1.16)	
Tsui et al., 2006 [72]	15.1%	HCV	ICD-9 code	In-hospital mortality: AOR 1.41 (95% CI 0.97–2.04)	8
				ALOS +19% (95% CI 12–27%)	
Wei et al., 2018 [73]	37.1%	HCV	ICD-9 code	Readmission within 30 days: AOR 1.14 (95% CI 1.08–1.19)	8
Wurcel et al., 2018 [74]	19.5%	HCV	ICD-9 code	Readmission within 30 days: AOR 1.73 (95% CI 1.31–2.29)	8

*Cross-sectional studies*					
Baum et al., 2008 [75] #	29.7%	HIV/HCV coinfection	HCV-RNA	Hospitalization: AOR 2.77 (95% CI 1.21–6.34)	3
El Khoury et al., 2012 [76]	50.0%	Untreated HCV	Self-reported	Annual hospitalizations: 0.42 vs. 0.25, *p*=0.07	4
Vietri et al., 2013 [77]	50.0%	HCV	Self-reported	Annual hospitalizations: 0.52 vs. 0.27, *p*=0.073	4

AAAPC: adjusted average annual percentage change; AHR: adjusted hazard ratio; AIRR: adjusted incidence rate ratio; ALD: alcohol-induced liver disease; ALOS: length of stay; anti-HCV: hepatitis C virus antibody; AOR: adjusted odds ratio; ARR: adjusted rate ratio; ART: antiretroviral therapy; ASHR: adjusted standardized hospitalization ratio; CI: confidence interval; DAA: direct-acting antiviral; HBsAg: hepatitis B virus surface antigen; HBV: hepatitis B virus; HCC: hepatocellular carcinoma; HCV: hepatitis C virus; HIV: human immunodeficiency virus; ICU: intensive care unit; NSS: not statistically significant; PI: protease inhibitor. †Analysis was carried out on *N* = 263062 (HIV/HCV coinfection 56304; HIV mono-infection 206758). ‡HCV 1.36% of total hospitalizations *N* = 79185729. Analysis and findings reported for the N and HCV% reported in Tables 1 and 2, respectively. §Does not describe coinfection with HBV. ¶HCV 0.47% in 1700400 total joint arthroplasties during the study period. Analysis was carried out on *N* reported in Table 1. #HCV 33.02% in original *N* = 218. Analysis was carried out on *N* reported in Table 1.

## Data Availability

The data that support the findings of this systematic are publicly available and included in the manuscript and supporting files.

## Supplementary Materials

Supplementary Materials S1 TABLE: search strategy in OVID Medline.
