# Supplementary material for: Hepatitis C Virus Infection and Hospital-Related Outcomes: A Systematic Review
Source: Can J Gastroenterol Hepatol. 2024 Mar 7;2024:3325609. doi: 10.1155/2024/3325609 (PMC10940031; doi:10.1155/2024/3325609)
Supplement: Supplementary Materials — S1 TABLE: search strategy in OVID Medline. [file 3325609.f1.docx]

**S1 TABLE: Search strategy in MEDLINE, EMBASE, CINAHL, PsycINFO, and Web of Science**

| **Number** | **Search statement** |
| --- | --- |
| 1 | exp Hepatitis C/ or Hepatitis C, Chronic/ or hepatitis c.mp. or HCV.mp. |
| 2 | hospitalization.mp. or exp Hospitalization/ or hospital*.ti. |
| 3 | length of stay.mp. or "Length of Stay"/ |
| 4 | Patient Readmission/ or readmission.mp. or re-admission.mp. |
| 5 | in-hospital mortality.mp. or Hospital Mortality/ or hospital mortality.mp. |
| 6 | Patient Discharge/ or discharge against medical advice.mp. or against medical advice.mp. |
| 7 | 2 or 3 or 4 or 5 or 6 |
| 8 | 1 and 7 |
